# Growing Yeast into Cylindrical Colonies

**DOI:** 10.1016/j.bpj.2014.02.040

**Published:** 2014-05-20

**Authors:** Clément Vulin, Jean-Marc Di Meglio, Ariel B. Lindner, Adrian Daerr, Andrew Murray, Pascal Hersen

**Affiliations:** † Laboratoire Matière et Systèmes Complexes, Centre National de la Recherche Scientifique and Université Paris Diderot, Paris, France; ‡ Institut National de la Santé et de la Recherche Médicale, Faculté de Médecine, Université Paris Descartes, Paris, France; § Molecular and Cellular Biology, Harvard University, Cambridge, Massachusetts; ¶ The Mechanobiology Institute, National University of Singapore, Singapore

## Abstract

Microorganisms often form complex multicellular assemblies such as biofilms and colonies. Understanding the interplay between assembly expansion, metabolic yield, and nutrient diffusion within a freely growing colony remains a challenge. Most available data on microorganisms are from planktonic cultures, due to the lack of experimental tools to control the growth of multicellular assemblies. Here, we propose a method to constrain the growth of yeast colonies into simple geometric shapes such as cylinders. To this end, we designed a simple, versatile culture system to control the location of nutrient delivery below a growing colony. Under such culture conditions, yeast colonies grow vertically and only at the locations where nutrients are delivered. Colonies increase in height at a steady growth rate that is inversely proportional to the cylinder radius. We show that the vertical growth rate of cylindrical colonies is not defined by the single-cell division rate, but rather by the colony metabolic yield. This contrasts with cells in liquid culture, in which the single-cell division rate is the only parameter that defines the population growth rate. This method also provides a direct, simple method to estimate the metabolic yield of a colony. Our study further demonstrates the importance of the shape of colonies on setting their expansion. We anticipate that our approach will be a starting point for elaborate studies of the population dynamics, evolution, and ecology of microbial colonies in complex landscapes.

## Introduction

The growth of microbial colonies on solid substrates is a fascinating, difficult-to-capture morphogenetic process (1–4). Growth morphologies range from the smooth, flat, conical colonies formed by laboratory strains to the much more complex shapes often displayed by wild-type strains (5,6). Colonies expand by consuming nutrients provided by their moist, solid substrate. They typically contain a few tens of millions of cells/mm^3^, that is, several million to a few billion cells for large colonies. In a well-mixed liquid culture, the single-cell division rate, *μ*, is the only parameter needed to model the exponential growth of the number of cells in the population. In contrast, in a colony, the number of dividing cells is unknown a priori. This number likely depends on the colony morphology and on the properties of the substrate (7) due to the establishment of local gradients of nutrients that set the distance over which nutrients can diffuse into the colony. Also, the presence of surrounding microbial colonies can add another layer of complexity and can alter the growth of the colony through the release of toxic chemicals into the environment (6,8). Therefore, it is not possible to infer the radial and vertical growth rates of a colony solely from *μ*. A key difference between a well-mixed planktonic culture and a colony is that the cells in a colony compete locally for nutrients. Cells buried deeply within the colony may be deprived of nutrients as the cells near the colony boundary can absorb the nutrients first (4,9). As a consequence, the cells within a colony experience different microenvironments, and their metabolic yield (number of cells produced per unit of nutrients), growth rate, and nutrient consumption depend on their position inside the colony (10,11). After several days of growth, colonies are thus composed of cells which have different properties and which can even show cellular differentiation depending on their position within the colony (12,13).

**Figure 1:**
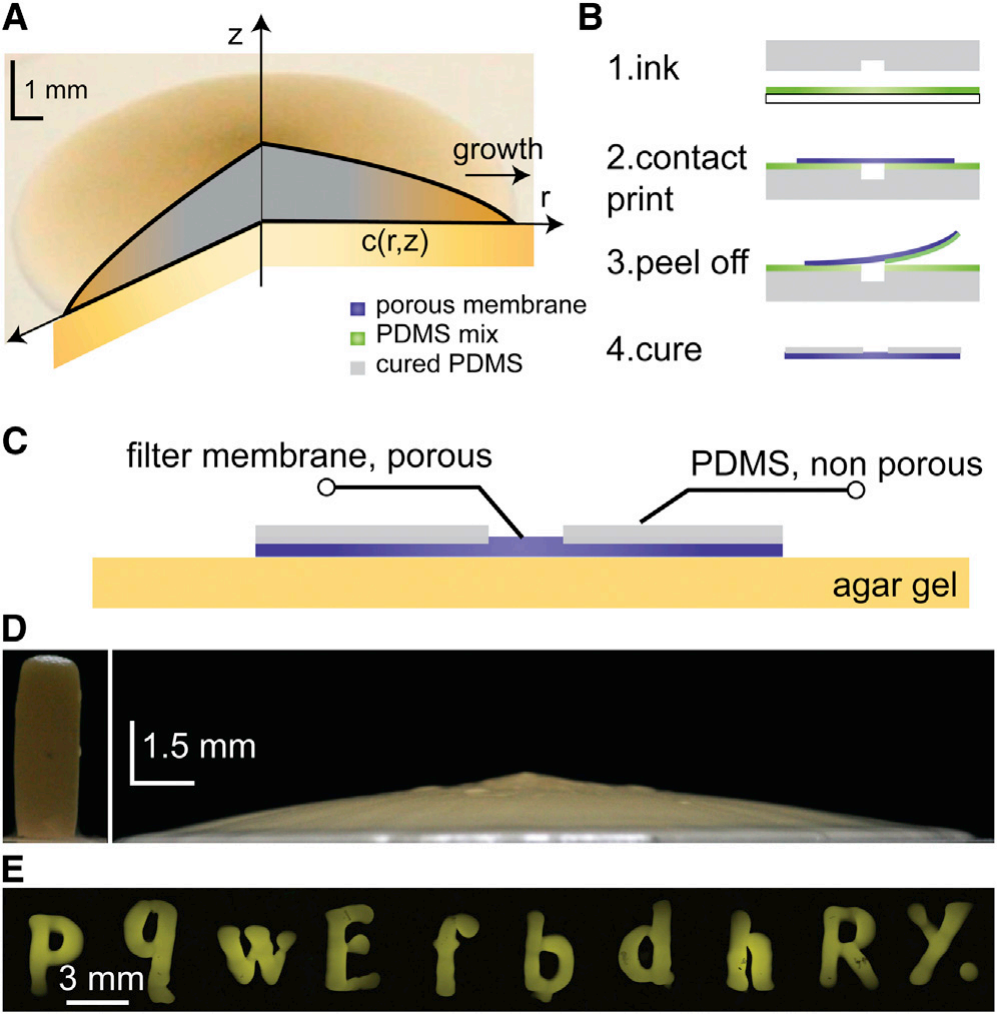
Yeast colonies can be grown into any geometrical shapes. (*A*) Yeast colonies growing on an agar gel usually display a smooth, flat conical shape that expands radially and vertically. Cells take up nutrients (present in the gel at concentration *C*(*r*,*z*)) and generate chemical gradients inside both the colony and the supporting agar gel. (*B*) To limit nutrient delivery to selected areas, we modified the porosity of a filter membrane by contact printing of PDMS. A stamp (*gray*) is inked using a thin film of liquid PDMS (*green*) (*1*). The stamp is used to contact print a porous filter membrane (*violet*) (*2–4*). The liquid PDMS enters the pores of the membrane at the printed locations and clogs them when cured (*4*). Any patterns of porosity with features larger than a few hundred micrometers are achievable. (*C*) Once placed on top of a solid culture medium (*yellow*), the patterned membrane defines the locations where nutrients can be delivered from the agar gel to an assembly of cells placed on the surface of the membrane. (*D*) On such systems, yeast colonies adopt shapes that faithfully respect the pattern of porosity. When grown on a porous disk, colonies grow as cylinders (*left*); the diameter of the cylindrical colonies remains constant while they grow in height (see also Movie S1). The aspect ratio of cylindrical colonies is thus very different from freely expanding colonies (*right*). (*E*) A yeast strain expressing a fluorescent protein (YFP) could be cultured into colonies shaped as letters of the alphabet with high fidelity using our method. To see this figure in color, go online.

Descriptions of nutrient diffusion within colonies are commonly derived from Pirt's model (9,14) which can be represented as a pile of cells expanding primarily at its edges where nutrients are available (Fig. 1 *A*), whereas at the center of the colony, nutrients are quickly exhausted and the local growth rate is close to zero (9,15). Pirt’s model predicts that the average radius of a colony increases linearly with time: only a fixed number of cells near the expanding edge of the colony have access to nutrients and can divide (Fig. S1 in the Supporting Material). Experimental data from growing colonies is usually focused on basic morphological measurements, and fit Pirt's prediction (14–17) by demonstrating a linear radial growth rate. However, the complexity of the varied growth rates within a colony does not allow the prediction of nutrient concentration profiles and thus prevents an assessment of the interplay between nutrient intake and expansion of the freely growing colony. This strongly limits our capacity to build a predictive model of the growth and metabolic state of three-dimensional colonies. Analytical models usually address the two-dimensional problem of an infinite biofilm plane or approximate colonies as flat, two-dimensional discs (14,16–18). Three-dimensional colonies still lack a proper mathematical description, and there is a need for a better experimental framework to study microbial colony growth and metabolism.

Here, we propose an experimental framework to study the growth of (*Saccharomyces cerevisiae* yeast) colonies. We first describe the fabrication and use of micropatterned porous membranes on top of which yeast colonies of any geometry can be grown. We then study the vertical growth of cylindrical yeast colonies in detail and show how, in this simple geometry, the yield of a cylindrical colony can be extracted from its vertical growth rate. Finally, we discuss the relevance of this method for microbiology.

## Materials and Methods

### Patterned membrane fabrication

We used contact printing to locally modify the porosity of commercially available filter membranes (19). A polydimethylsiloxane (PDMS) stamp was first created by either micromachining or soft lithography. A fresh PDMS mixture (Sylgard 184, Dow Corning; or alternatively black PDMS, Sylgard 170) of the pre-polymer and its curing agent was prepared in a 1:10 ratio, degassed and spread evenly onto a flat, clean surface to obtain a thin layer. The PDMS stamp was inked by placing it on top of the PDMS mixture layer. The stamp was then placed upside down, and a porous filter membrane was placed in contact with the stamp, withdrawn, and cured for 1 h at 80°C with the patterned side face-up. In this study, we mainly used Isopore (0.22 *μ*m) filters from Millipore, but we have also successfully modified other types of filter membranes, including Anodisc (0.022 *μ*m, 0.1 *μ*m, 0.22 *μ*m), Cyclopore (0.22 *μ*m), and Nuclepore (0.22 *μ*m), from Whatman (Maidstone, United Kingdom).

### Yeast strains and inoculation

*S. cerevisiae* cells were cultured overnight in liquid yeast extract peptone dextrose (YPD) (1% yeast extract, 2% peptone, 2% glucose) medium at 30°C, then diluted 100-fold into fresh medium and incubated for 4 h at 30°C to reach the exponential growth phase. Membranes were sterilized with ultraviolet light (30 min) on both sides. A membrane was placed on top of a flat YPD agar gel, and drops of yeast were deposited onto the porous areas. The inoculated plates were incubated overnight to allow the drops to dry and then flipped upside down to prevent gravity from deforming the yeast colonies. All experiments were performed at 30°C using a BUD4, haploid, prototrophic derivative of W303 yeast strain.

### Glucose dosage

Glucose concentrations were assayed using a specific enzymatic oxidation method coupled to NAD reduction (GAHK20; Sigma Aldrich, St. Louis, MO) according to the manufacturer’s instructions. Briefly, filter weighing paper (#531; VWR) was cut into 0.75 mm-radius discs, and single disks were placed between the agar gel and membrane before inoculation. After 1 week of growth, the filters were picked up, soaked in 20–180 *μ*L dosing solution, and the absorbance value of the supernatants at 340 nm was read after 1 h incubation and compared to a calibration curve created using filters placed far from the colony or in colony-free gels.

### Image acquisition and analysis

Pictures were acquired using a Canon (Tokyo, Japan) EOS 400D camera. Images and movies were analyzed with ImageJ (20) (http://rsbweb.nih.gov/ij/). Colony dimensions were measured by comparison to the dimensions of a scaling object placed next to them.

### Numerical computation

Numerical modeling was implemented using COMSOL v3.5 (COMSOL, Stockholm, Sweden). We solved the axis-symmetrical diffusion equation with absorption in a preformed cylindrical colony. The model implementation is detailed in the Supporting Material.

## Results

### Yeast colonies can be grown in the desired shape by controlling the location of nutrient delivery

When grown on a flat agar surface, yeast colonies appear as flattened cones that expand radially. We prevented this lateral expansion using an inexpensive and versatile culture system to control the location of nutrient delivery to an assembly of yeast cells. We obstructed the pores of a commercially available filter membrane at selected locations using contact printing (21) of a thin layer of PDMS (Fig. 1 *B*). The membranes retained their initial porosity everywhere except where PDMS was deposited and cured to clog the pores of the filter membrane (Fig. 1 *C*). The fabrication process described in the Methods section allowed a printing resolution of typically 200 *μ*m (Fig. 1 *A*). Membranes were then placed onto the surface of an agar gel containing nutrients, and yeast cells were inoculated onto the porous areas of the membrane, through which they could access nutrients through the pores of the filter membrane. Strikingly, yeast colonies grew vertically as if extruded from the porous areas (Fig. 1, *D* and *E*). Several days of growth resulted in high-aspect-ratio colonies (typically 4:1 for a cylindrical colony 1.5 mm wide and 2 weeks old), in contrast to the classic flat morphologies (Fig. 1 *D*). Moreover, the shapes of the yeast colonies remained similar to the initial pattern of porosity over several days - contrary to previous attempts at bacterial printing (22). This concept is illustrated in Fig. 1 *E*, in which we forced yeast colonies to adopt the shape of letters of the alphabet.

### The growth rate of cylindrical colonies quickly reaches a constant value

**Figure 2:**
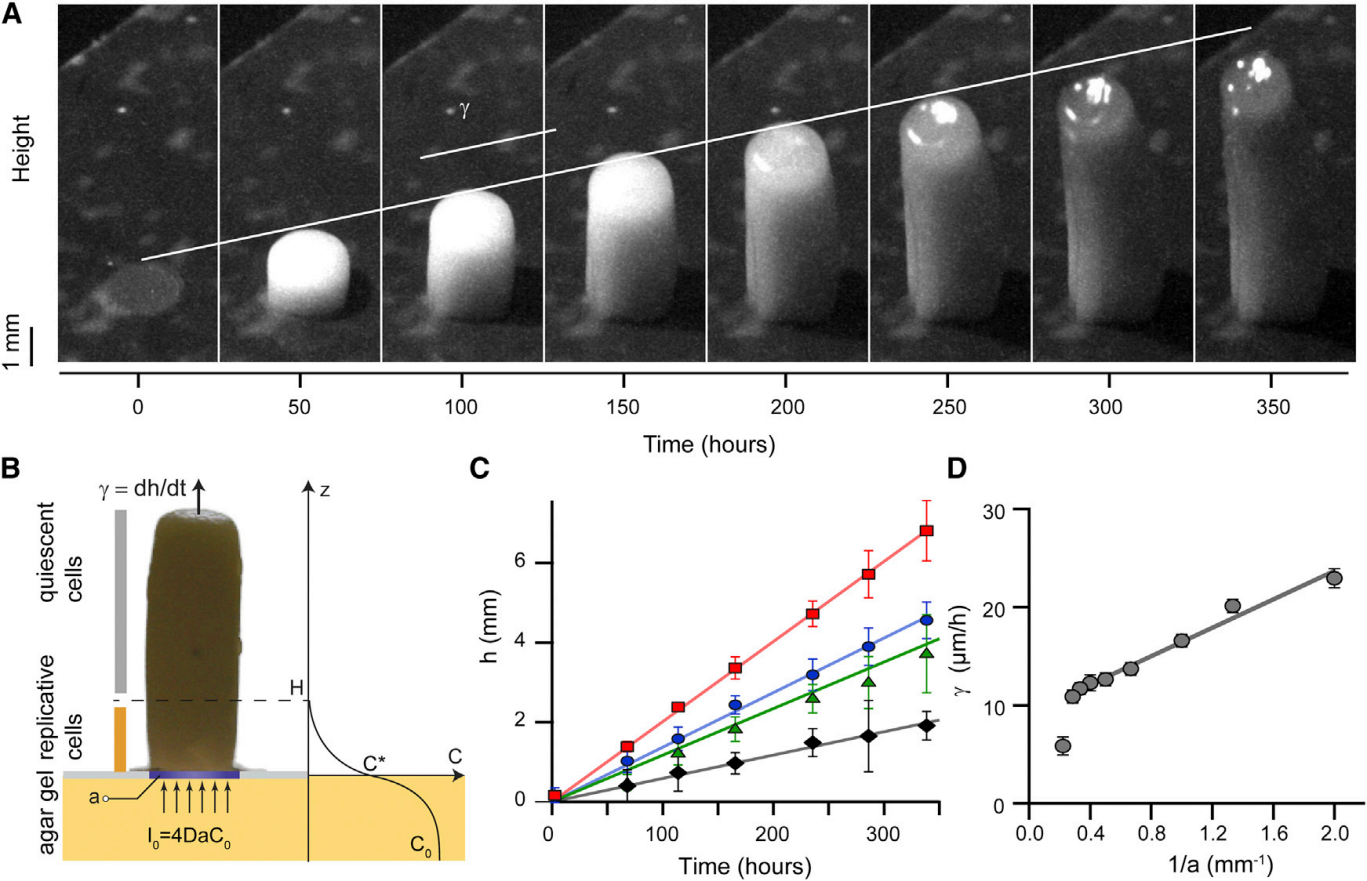
Yeast cylindrical colonies grow vertically at a constant rate that is inversely proportional to their diameter. (*A*) Time-lapse imaging of a cylindrical yeast colony (1.5 mm in diameter) grown on 2% glucose. The cylindrical colony grows vertically, with no lateral expansion. When performing long time-lapse cultures, the top part of the cylinder eventually dries out due to the absence of a lid covering the plate. This was not the case when imaging occasionally to obtain quantitative measurements of the growth rate (see *C*). (*B*) Sketch of a cylindrical colony. The vertical growth rate, *γ* = *dh/dt*, depends on the number of cells that have access to nutrients; such cells are located at the bottom of the cylindrical colony and set up a vertical gradient of glucose within the colony. We defined *C*_0_, the glucose concentration in the gel; *C^∗^*, the glucose concentration at the bottom of the cylindrical colony; *H*, the distance to which glucose diffuses inside the colony; and *a*, the radius of the yeast colony (see also the Supporting Material). The maximum influx of glucose that the agar gel can deliver is *I*_0_ = *4DaC*_0_ in the case of perfect glucose absorption at the base of the colony. (*C*) Thin cylindrical colonies grow faster than thick cylindrical colonies. Each data point represents the average values for (at least) four experiments; the straight lines are linear fits of the data (error bars represent the standard deviation). The diameters of the yeast cylindrical colonies were 1.5 mm (*red squares*), 3 mm (*blue circles*), 6 mm (*green triangles*), and 9 mm (*black diamonds*). (*D*) As expected from a simple model (see text), the vertical growth rate, *γ*, is inversely proportional to the radius, *a*, of the cylindrical colony. To see this figure in color, go online.

When the porous areas were disk-shaped, the yeast colonies grew as cylinders (see Figs. 1 *D* and 2 *A* and Movie S1). The reproducibility of the growth rate and morphology of these colonies contrasted with the variability of the exotic yeast stalks previously described by Engelberg et al. (23). The vertical growth rate of the cylindrical colonies was constant (≈20 *μ*m h^−1^ for 1.5-mm-diameter cylindrical colonies grown on 2% glucose) over several days (Fig. 2 *A*). This indicates that the system reached a steady state during which the number of cells dividing per unit time remained constant (Fig. 2, *B* and *C*). Close to the top of the colony, the cells displayed the physiological characteristics of starved, nonreplicating cells; however, these cells quickly resumed their growth if they were placed in contact with nutrients. Given the simple geometry of cylindrical colonies, the available nutrients are very likely to be consumed by a thin layer of cells located at the base of the cylinder (Fig. 2 *B*), whereas the upper cells have restricted access to nutrients and do not contribute to the growth of the colony. A simple guesstimate indicates that eight layers of 4-*μ*m-wide cells dividing every 90 min—the typical duration of the yeast cell cycle in liquid culture—would indeed lead to a vertical growth rate of ∼20 *μ*m h^−1^.

### The growth rate is inversely proportional to the radius of the cylindrical colony

As a first approximation, the rate of steady-state glucose influx at the base of the cylindrical colony can be expressed as $I_{0}=4DC_{0}a$ (24), where the colony is approximated as a fully absorbing disk of radius *a* placed on top of a semi-infinite space; $C_{0}$ is the glucose concentration, and D is the diffusion coefficient of glucose in the gel (Fig. 2 *B*). In contrast with a freely growing colony, the imposed geometry allows a simple quantitative estimate of the glucose influx and thus provides a method to relate colony growth to glucose consumption by the colony. The vertical growth rate of the cylindrical colony can then be expressed as $\gamma \equiv dh/dt=Y\left(I_{0}/\pi a^{2}\right)=\left(4DC_{0}/\pi a\right)Y$, where Y is the yield of the colony, defined as the volume of cells produced per unit of glucose consumed (see details in the Supporting Material). This predicts that the vertical growth rate, γ, scales as 1/a, in agreement with our experimental observations (Fig. 2 *D*). If the colony yield, *Y*, does not depend on the glucose concentration, then this analytic expression also predicts that the growth rate should increase linearly with the glucose concentration, $C_{0}$. On the basis of this hypothesis, any deviation from this linear dependency could then be attributed to variations in the colony yield with glucose concentration.

### The growth rate of vertically growing cylindrical colonies becomes saturated at high glucose concentrations

**Figure 3:**
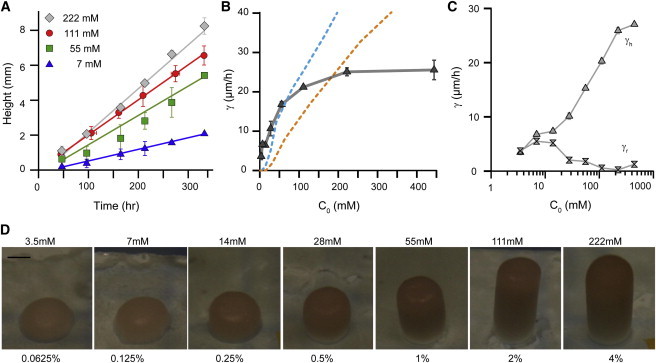
The growth rate of yeast cylindrical colonies increases with the glucose concentration. (*A*) Measurements of the height of cylindrical colonies as a function of time for different glucose concentrations (*C*_0_ = 7 mM (*blue triangles*); *C*_0_ = 55 mM (*green squares*); *C*_0_ = 111 mM (*red circles*); *C*_0_ = 222 mM (*black diamonds*)). (*B*) The experimental growth rate increases with the glucose concentration and becomes saturated around *C*_0_ = 200 mM (*gray triangles*). Numerical computations of the growth rate based on data from the literature do not fully capture the variation of the growth rate with the glucose concentration (*cyan dashed line*; data from Youk and van Oudenaarden (26); *orange dashed line*, data from Reifenberger et al. (27)). Note that we did not adjust any parameters to plot these curves (see the Supporting Material). None of the numerical models are able to capture the saturation of the vertical growth rate at higher glucose concentrations. (*C*) For low glucose concentrations (*C*_0_ < 10 mM), we observed both vertical, *γ*_h_, and radial expansion, *γ*_r_, at similar rates. This can be explained by the fact that under such conditions, cells metabolize glucose using oxygen, and thus growth at the periphery, where oxygen is present, is favored. (*D*) Aspect ratio of yeast cylindrical colonies as a function of the glucose concentration after 1 week of growth. Pictures were taken with the same magnification; the pattern diameter is 1.5 mm. To see this figure in color, go online.

To investigate the effect of nutrient availability on the vertical growth rate, we measured the growth rates of cylindrical colonies with a fixed diameter of 1.5 mm over a range of glucose concentrations. We found that the growth rate increased with the glucose concentration and reached saturation at a concentration of ∼200 mM (3% glucose; Fig. 3 *A*). This plateau is in agreement with the plateau observed for freely growing colonies (see Fig. S1). Interpreting this saturation is not straightforward. Increasing the glucose concentration should allow glucose to diffuse further into the colony, thus increasing the number of dividing cells and the vertical growth rate of the cylindrical colony. One possible explanation for the saturating glucose concentration is that under saturating concentrations, glucose may no longer be the limiting nutrient. We ruled out this hypothesis, as an increase in the concentrations of other nutrients did not prevent this plateau in the growth rate (see Fig. S2). We hypothesize that saturation of the growth rate could be due to an increase in the concentration of toxic metabolic waste, the result of cellular differentiation within the pillar, or the fact that single-cell yield decreases because ATP production is no longer the limiting factor for cell division.

To investigate the nutrient dependence of the growth rate, we implemented a numerical model for the vertical growth of a cylindrical colony. The model predicts the variation of the cell division rate, *μ*(*C*), and the specific absorption of glucose, *q*(*C*), with the local glucose concentration based on data from the literature (see details in the Supporting Material, Fig. S5, and Tables S1 and S2). Solving this model was simplified by the cylindrical geometry of the colony and, in particular, by the absence of lateral expansion of the colony. We used data from the literature for cells grown in liquid aerobic culture to solve the nonlinear diffusion equation, with diffusion of glucose inside the agar gel and inside the vertical colony, and absorption of glucose inside the colony. The growth rate computed by the numerical models did not show any saturation, despite the inclusion of a saturation of division rate and yield. This is consistent with our interpretation that the distance diffused by glucose inside the colony increased with glucose concentration, as did the vertical growth rate. Although the metabolic constraints that lead to saturation are not known and are beyond the scope of this study, our results show that a simple model in which a higher glucose concentration leads to more growing layers and a higher growth rate turns out to be incorrect.

### The yield of cylindrical colonies decreases as the external glucose concentration increases

**Figure 4:**
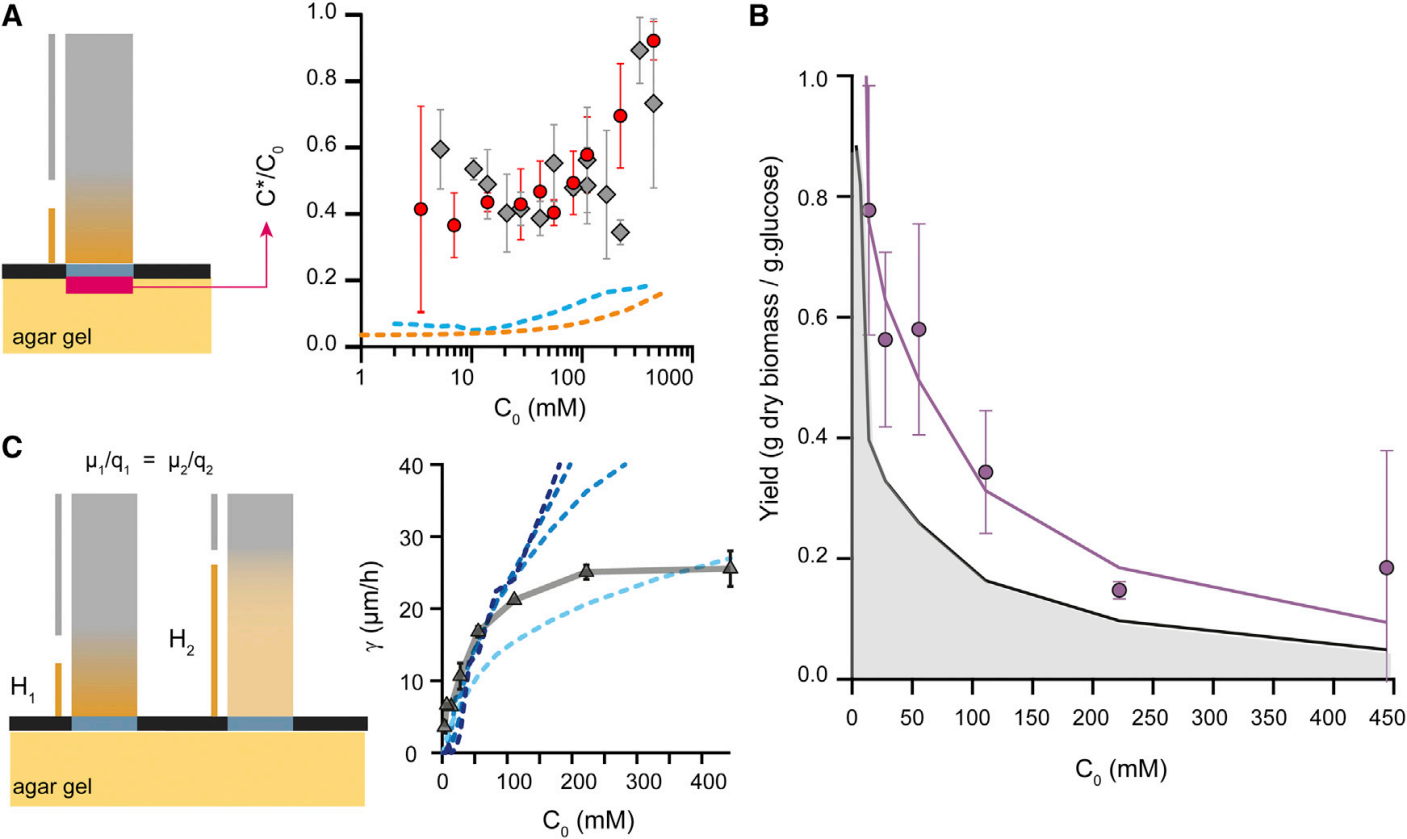
The growth rate of a cylindrical colony provides a direct estimate of the colony yield. (*A*) We defined the glucose concentration immediately below the colony as *C^∗^*. A disk of filter paper was placed below the colony, the colony was allowed to grow, and the concentration of glucose inside the filter paper was then assayed to determine *C^∗^*. The experimental values were noisy but typically gave a surface concentration of ∼40% of *C*_0_ (as measured on SC medium (*red circles*) or on YPD medium (*gray diamonds*)). A numerical estimate of *C^∗^* gave a much lower value of ∼10–20% of *C*_0_ (curves computed using data from Youk and van Oudenaarden (24) (*cyan dashed line*) and Reifenberger et al. (25) (*orange dashed line*)). (*B*) We measured the growth rate of several cylindrical colonies and determined glucose consumption as *4Da*(*C*_0_*− C^∗^*). We also plotted the minimum yield (*gray field area*) obtained at the same growth rate but under maximum glucose influx conditions of *4DaC*_0._ Yields were calculated based on measurements of 1-mm-high, 1.5-mm-diameter cylindrical colonies, equivalent to 1 mg of dry mass (see Fig. S4). (*C*) From an analytical perspective, we expect, to a first approximation, that the growth rate is primarily set by the yield of a colony, defined as the ratio of the average cell-division rate to the average glucose-absorption rate. To test this, we simultaneously varied both the cell division rate, *μ*, and the glucose uptake rate, *q*, obtained from Youk and van Oudenaarden (24). From light blue to dark blue, *q* and *μ* were both multiplied by 0.1, 0.5, 1, and 2. The curves collapsed into each other, demonstrating that, to a first approximation, the ratio of parameter *μ* to parameter *q* sets the dynamics of colony expansion. To see this figure in color, go online.

By combining the measured growth rate with an estimate of maximal glucose influx into the colony, we can make a noninvasive estimate of the yield of the cylindrical colony. Using the experimental growth rate of 21 *μ*m h^−1^ for a 1.5-mm-diameter disk on a rich glucose environment ($C_{0}$ = 111 mM) and the maximal analytical flux *I* gives a lower limit of the yield of *Y*_0_ = 0.16 g biomass/g glucose. This value is between the yield obtained in liquid chemostat for anaerobically-fermenting yeast cells (0.1 g biomass/g glucose) and respiring yeast cells (0.5 g biomass/g glucose) at a dilution rate of 0.1 h^−1^ and a relatively low steady-state glucose concentration of 0.2 mM (25). Fig. 4 *B* shows the variation in the yield over a range of glucose concentrations based on the vertical growth rate and our analytical estimate of glucose consumption. As a consequence of saturation of the growth rate at high glucose concentrations, the yield of cylindrical colonies decreased as the glucose concentration increased.

### The glucose influx rate depends on the colony uptake capacity

We previously assumed a glucose concentration of *C* = 0 at the bottom of the colony leading to a total glucose absorption. This would mean that only the most outer layer of cells could grow. This does not fit well with our previous estimate of eight layers of cells participating in the vertical growth of the colony. We improved our analytical model by introducing *C^∗^*, the surface concentration of glucose at the base of the colony (Figs. 2 *C* and 4 *A*). In this case, the nutrient flux absorbed by the cylindrical yeast colony can be represented as $I=4Da\left(C_{0}-C^{*}\right)$: this value is lower than that for the ideal case of a perfectly absorbing disk. The surface concentration of glucose is a signature of the metabolic activity of the cylindrical yeast colony and, as such, is an important parameter for describing the growth of a colony (see the Supporting Material).

We measured *C*^∗^ by placing small disks of filter paper below the colonies and assaying the glucose concentration inside the disks (see methods and Fig. 4 *A*). The filter paper had minimal effect on the growth rate of cylindrical yeast colonies: the vertical growth rate of colonies with filters was typically 90% of the vertical growth rate of cylinders without the filter (see Fig. S3). For *C*_0_ > 3 mM, we found that the surface concentrations of glucose were relatively high, and in the order of at least 40% of the $C_{0}$ (see Fig. 4 *A*). For $C_{0}$ > 111 mM, the measurements were not fully reliable, due to the formation of gas bubbles between the filter paper and the porous membrane; these bubbles were not observed in the absence of the paper. The glucose concentration at the bottom of the cylindrical yeast colonies was more than twofold lower in the numerical simulations than in our experimental measurements. This suggests that the cells were dividing more slowly in the experiments, thus consuming less glucose per unit time, allowing glucose to diffuse a greater distance inside the colony. This implies that the glucose concentration was higher at the interface and that the glucose influx thus was smaller (see the Supporting Material) than in our numerical simulations. Note that these simulations were based on experimental data from the metabolic behavior of cells in well-mixed liquid cultures—which may not provide a faithful representation of what occurs inside a multicellular assembly (Fig. 4 *B*). Indeed, and as we explained previously, yeast cells composing old colonies show cellular differentiation depending on their spatial position (13). Using the experimental growth rate of 21 *μ*m h^−1^ for a 1.5-mm-diameter disk on a rich glucose environment ($C_{0}$ = 111 mM), and taking into account the correction for the glucose surface concentration, gives *Y* = 0.34 g biomass/g glucose. Fig. 4 shows the experimental yield of a cylindrical yeast colony over a range of glucose concentrations.

## Discussion

In this work, we describe a method used to physically constrain the growth of yeast colonies. This simple and versatile method enabled us to reformulate a complex three-dimensional problem into a one-dimensional problem, allowing us to establish a simple analytical model for the vertical growth rate that revealed that cylindrical colonies grow more slowly than expected at a high glucose concentration. The cylindrical colony geometry that we studied, with its pattern of vertical expansion, is an ideal candidate for experimental and theoretical study of the trade-off between the single-cell growth rate (*μ*) and the glucose absorption rate of single cells (*q*) with respect to the colony growth rate. As an example, consider two colonies formed by cells with a similar yield (*μ/q*) but different growth rates (*μ*1 > *μ*2) and specific glucose absorption rates (*q*1 > *q*2). The specific glucose absorption rate defines the distance to which glucose can diffuse inside the colony, and therefore determines the number of dividing cells. The cell cycle duration sets the doubling time of the cells. Since the vertical growth rate depends linearly on the yield of the colony in the first approximation, one can predict that both colonies will have the same vertical growth rate (see Fig. 4 *C*). This prediction holds as long as the specific glucose absorption rate, *q*, is larger than the typical value, $\tilde{q}=8DC_{0}/\pi^{2}a^{2}$, at which the colony can be approximated as fully absorbing (see the Supporting Material). Usual measurements in aerobic liquid cultures (26,27) would give a specific glucose uptake rate of ∼50 × $\tilde{q}$ for *C*_0_ = 111 mM. This would mean that the colonies behave as fully absorbing. Note, however, that measured *C*^∗^ are higher than model predictions, suggesting that planktonic single-cell behavior is different from that of cells within a multicellular structure. The effect of both growth rate and uptake is thus of prime importance in understanding the expansion of spatially structured colonies. Indeed, previous numerical simulations have shown that a structured microenvironment could provide a fitness advantage to slow-growing strains (28–30); we anticipate that our growth method could be used to study this observation experimentally. It is important to note that recent studies (12,13) have shown that yeast cells undergo cellular differentiation within a colony linked to the stratification of the colony into two regions, the top and the bottom of the colony. This is an important aspect of the emergence of a spatial structure inside a colony, and it would be interesting to study this experimentally for cylindrical colonies and for various glucose concentrations to see to what extent this spatial organization may explain the saturation of the cylindrical-colony growth rate observed at high glucose concentrations.

Further studies should focus on the link between the colony shape and its metabolism. At very low glucose concentrations, the cylindrical colonies slowly expanded laterally over the printed pattern (Fig. 3 *C*). This observation can be understood by the fact that cells growing in low glucose conditions are respiring glucose and thus require oxygen. There is likely no oxygen in the center of the colony, as suggested by the absence of fluorescent protein maturation inside the colonies (see Fig. S2). Moreover, the ability of yeast colonies to grow under anaerobic conditions shows that respiration is not needed for the growth of cylindrical colonies. However, glucose respiration can occur on the periphery of the colony - assuming that the glucose is not consumed first by fermentation in the center of the colony. As shown in Fig. 3, radial expansion only occurred below *C^∗^* ∼ 6 mM (*C*_0_ ∼ 10 mM). As a result, we observed slightly rounder colonies in very low glucose environments (Fig. 3 *D*). Forcing yeast to grow in an argon atmosphere prevented such lateral expansion (Fig. S2), suggesting the existence of a metabolic switch at the level of the colony similar to the Crabtree effect observed at the single-cell level. This also suggests that for glucose concentrations above this threshold, the growth of cylindrical yeast colonies is primarily achieved by fermentation and not by respiration. Indeed, we obtained the same growth rates on 2% glucose medium when growing yeast in an argon atmosphere (see Fig. S2 *D*).

## Conclusion

Recent investigations have shown that both glucose sensing and uptake are essential for setting the division rate of single cells (26). We believe that these effects are also at play on the scale of a colony, where individual cells experience a range of glucose concentrations and are likely to express a variety of glucose transporters. More generally, our study sheds light on the differential effect of cell division and glucose uptake in setting the growth rate of well-mixed cultures and spatially structured cell assemblies; we argue that these differences should be taken into account when discussing population dynamics for colonies grown on solid substrates. By exploring the full range of growth geometries, we envision that our experimental framework can be used to quantitatively investigate the relationship between colony shape and colony fitness, as well as to evaluate several unresolved issues of population dynamics and microbial ecology, including cooperation and competition between different strains and adjacent colonies (31,32).

## Supporting Material

Document S1. Figs. S1–S6, Tables S1–S2, details of the analytical model, and references (33–37)

Movie S1. Growing Yeast into Cylindrical Colonies A *S. cerevisiae* colony is grown on a modified Isopore membrane patterned with a 1.5-mm-diameter porous disk. The colony develops upside down (to avoid buckling) for over two weeks with a constant growth rate. Snapshots of this video are shown in Fig. 2 *A*.

Document S2. Article plus Supporting Material
