# Supplementary material for: Growing Yeast into Cylindrical Colonies
Source: Biophys J. 2014 May 20;106(10):2214–21. doi: 10.1016/j.bpj.2014.02.040 (PMC4052359; doi:10.1016/j.bpj.2014.02.040)
Supplement: Document S1. Figs. S1–S6, Tables S1–S2, details of the analytical model, and references (33–37) [file mmc1.pdf]

Supplementary Information

Clément Vulin[1], Jean-Marc Di Meglio[1], Ariel Lindner[2], Adrian Daerr[1], Andrew Murray[3] and Pascal Hersen[1,4,*]

[1] Laboratoire Matière et Systèmes Complexes, UMR 7057 CNRS & Université Paris Diderot, 10 rue Alice Domon et Léonie Duquet, 75013 Paris, France.

[2] INSERM U1001, Faculté de Médecine, Université Paris Descartes, 24 rue du Faubourg Saint Jacques, 75014 Paris, France.

[3] Molecular and Cellular Biology, Harvard University, Cambridge, MA 02138, USA.

[4]The Mechanobiology Institute, National University of Singapore, 5A Engineering Drive 1, Singapore 117411, Singapore.

*Correspondence and requests for materials should be addressed to Pascal Hersen (pascal.hersen@univ-paris-diderot.fr).

1. Supplementary Figures

Figure S1

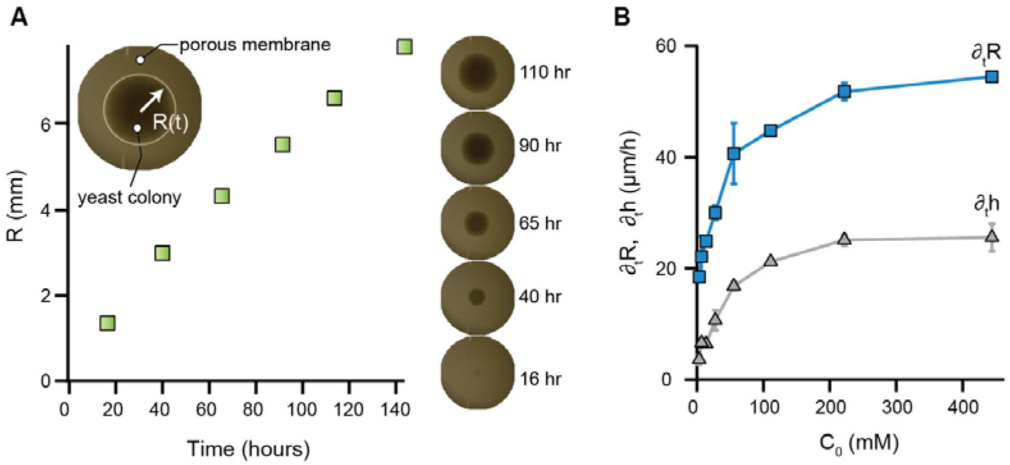

Figure S1.The radial growth of yeast colonies on an unmodified filter membrane. A. A colony of *S. cerevisiae* is grown on a porous membrane placed on top of an agar gel (YPD, 2% glucose = 111 mM). The radius of the colony, *R*, increases linearly with time at a rate of nearly 50µm/hr. **B.** Freely growing colonies (blue squares) exhibits a saturation of the radial growth rate with increasing glucose concentration as observed for the vertical growth of cylindrical yeast (grey triangles, a=0.75 mm). All error bars are standard deviations, n>3.

Figure S2

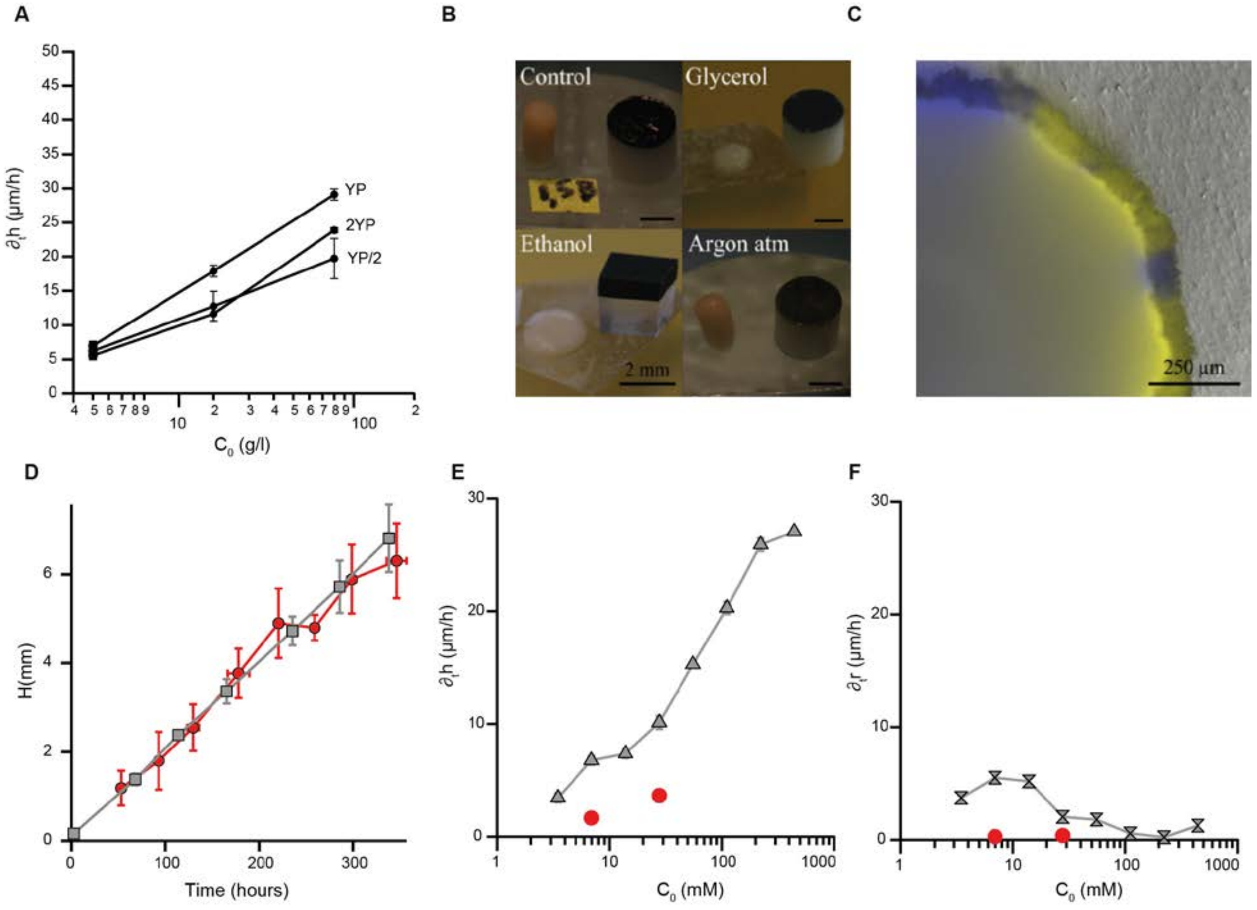

Figure S2. Some metabolic properties of cylindrical colonies. A. The variation of the vertical growth rate with glucose is preserved when the yeast extract and peptone concentrations are halved or doubled in the medium. **B.** Pictures of 1 week old colonies when grown on 2% glucose, 3% glycerol or 3% ethanol as non-fermentable carbon source, and in anoxic conditions (argon atmosphere). Colonies that develop on non-fermentable carbon source did not grow vertically and spread horizontally, suggesting that growth can only occur where they have access to oxygen. The black topped objects are vertical scale markers. **C.** A cylindrical colony seen from below. Cells of the colony were constitutively expressing either YFP or CFP. Fluorescence was visible only at the edge of the colony, which is consistent with the absence of fluorophore maturation under anoxic conditions, and hence suggests that there is little available oxygen inside the colony. **D.** The vertical growth rates of colonies grown on 2% glucose and under argon atmosphere (red, no oxygen) or in air (gray) are similar. This suggests that on high glucose, the yeast cylinder grow primarily by fermentation. **E.** This is further confirmed by looking at the growth at low glucose under argon atmosphere. The residual vertical growth (circles) rate is smaller in presence of oxygen (regular growth, grey triangle) indicating that both respiration and fermentation takes place at low glucose and participate to the yeast cylinder rise. **F.** Moreover, the radial growth rate becomes very close to zero and the shape of the yeast colonies is closer to that of a cylinder, as expected from a pure vertical growth of the colony.

Figure S3

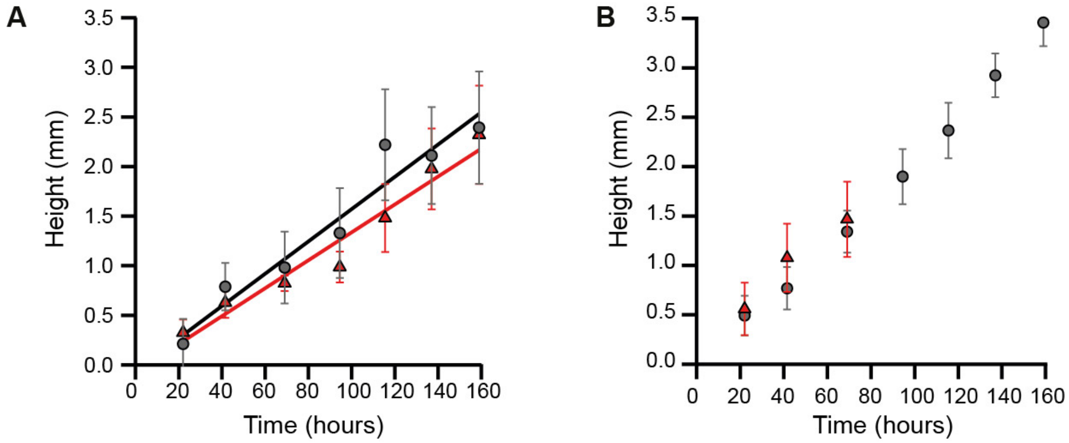

Figure 3: The growth of cylindrical yeast colony is not altered by intercalation of a disc of filter paper. We compared the growth of vertical colonies on 2% glucose in the absence (grey circle) or presence (red triangle) of a thin disk of filter paper that we used to measure the surface glucose concentration below the colony. A. For 1% glucose (C_0 = 55mM), the growth rates were comparable (the slopes of the linear fits were within 13%, which is less than the sums of the standard deviations of 8% (filter) and 11% (control)). B. For 2% glucose (C_0= 111mM), the filter paper did not alter the growth rate for the first 100 hours.

Figure S4

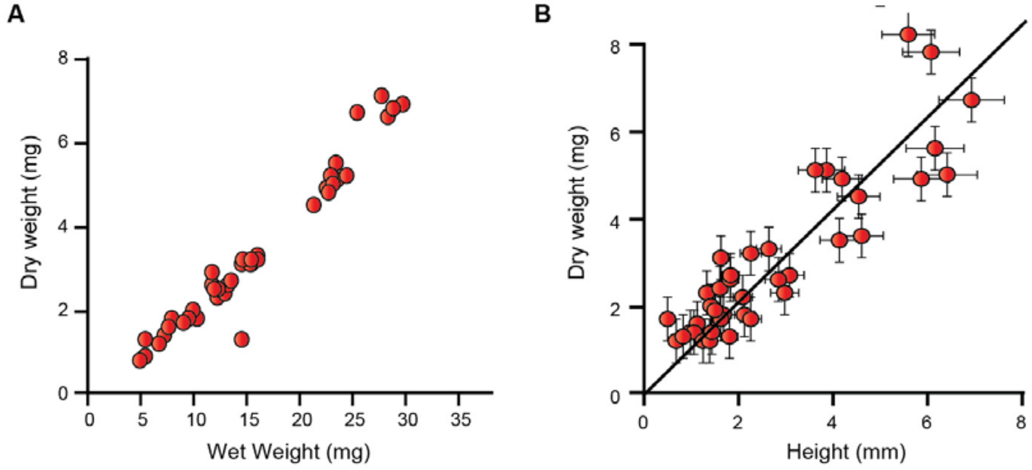

Figure S4: We measured the wet mass, dry mass and height of yeast cylindrical colonies of 1.5 mm diameter. Results show a linear relation between those three quantities. Using linear regression we found that there is 1 mg of dry mass per mm height of a 1.5 diameter cylindrical colony. **A.** Dry weight (mg) *vs.* wet weight (mg). **B.** Dry weight (mg) *vs.* height (mm). The slope is 1 mg/mm if the fit is forced to pass by the origin (Pearson coefficient of 0.89). We used this to convert our measurement of cylindrical vertical size into a biomass.

Figure S5

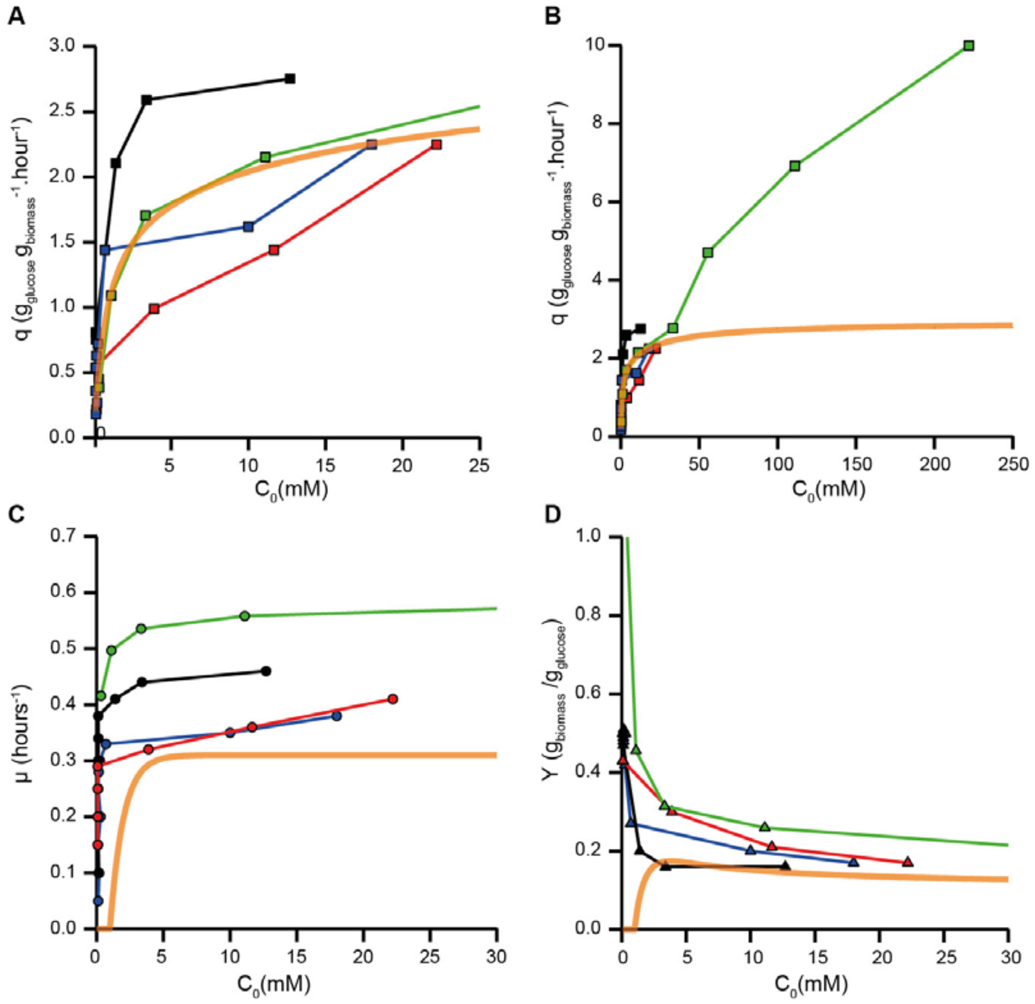

Figure S5: Division rate and glucose specific absorption measurements from the literature. (A, B) Glucose absorption, *q*, **(C)** division rate, *μ*, and **(D)** the yield, Y, are shown as a function of the glucose concentration of the medium in which cells were cultured. Interpolated data from Youk *et al.*, (green) are shown along with data from 3 chemostats measurements: red (1), black (2), blue (3). The pale orange curves are adapted from Reifenberger *et al.* (4) **(see Table S7)**. The panel **B** shows *q(C)* for higher concentration as measured by Youk *et al*. (5) Note that original data were not given in comparable units and we had to convert them. This was done by using data from the literature (6) for the cell volume (35 μm^3), the cell density (1.1 g/mL) and the cell dry weight (40% of its wet weight). Although there are some quantitative differences, the qualitative variations are comparable between the different sets of data.

Figure S6

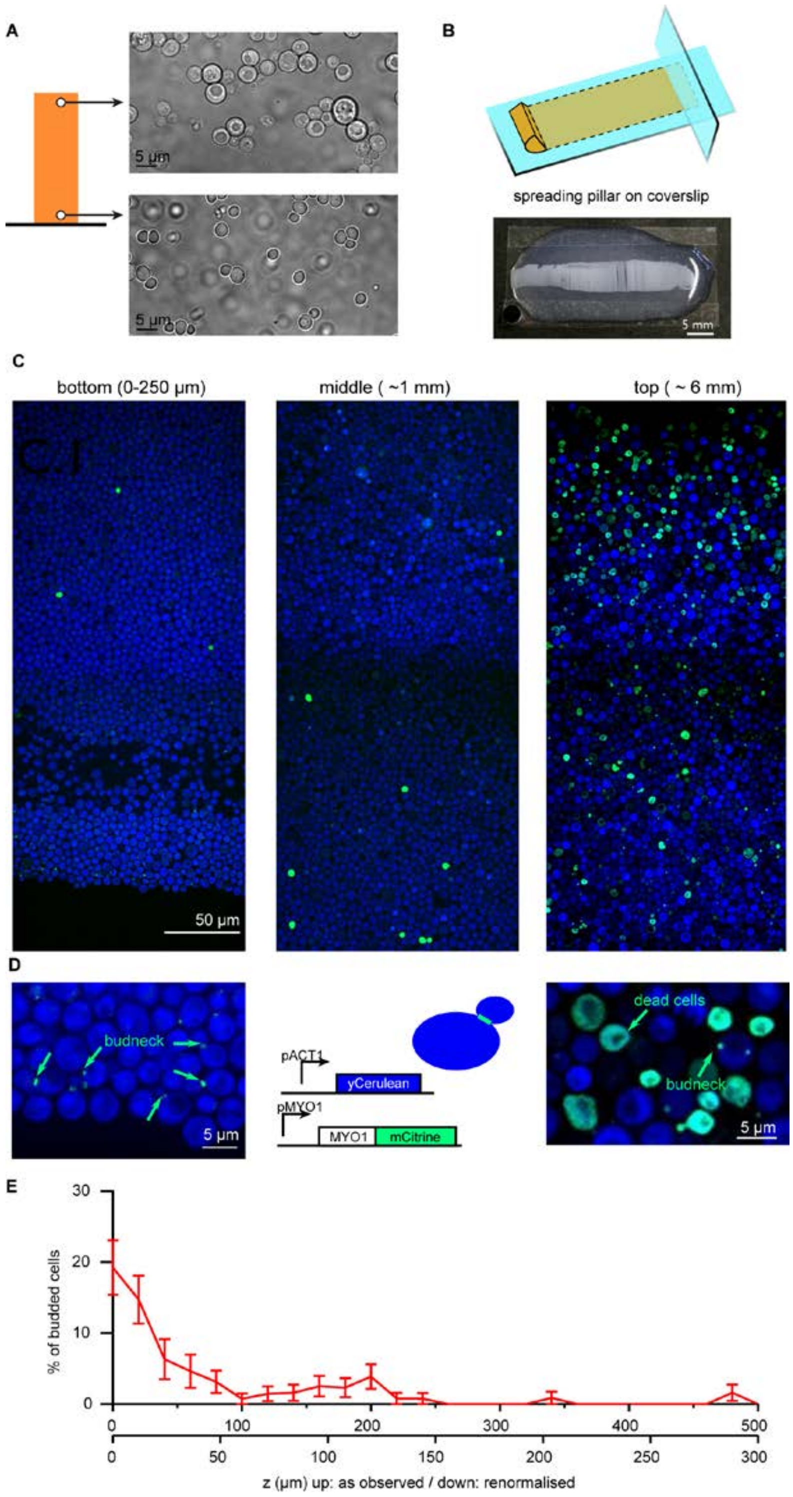

Supplementary figure S6: Microscope analysis of colony. **A.** Transmission light microscopy images of 2 samples taken in a live colony, respectively at the top and close to the membrane. Cells at top show physiology of stressed cells, with big volume, important vacuoles, or apparition of irregular filling of the cell, and probably dead cells (see C, D). Cells at the bottom showed more regular shape and several buds (see D). **B.** Description of protocol used to image cells at their position. A colony was tilted to the side, separated from the membrane and spread on a coverslip. A layer of 40°C low melt agar was quickly poured on top and the colony was imaged after 1 hour, to avoid inhomogeneous fluorescent protein folding that could occur in the low oxygen environment of colony core. **C.** Confocal (spinning disc) images of the spread colony were taken at different positions (bottom, middle and top). **D.** At the bottom of the colony, ~20% of cells showed budnecks (left). This was not the case for upper layers (see right). Center: Yeast strain constitutively expressing yCerulean and with MYO1 tagged (mCitrine) protein was used to spot cell division. MYO1 localizes at bud site early in cycle and stays there until division is completed. **E.** Proportion of cells showing the MYO1 ring decreases with upward position in colony. The spreading of pillar on coverslip didn't allow for proper quantitative positioning but an approximate renormalization in the z direction is shown as a lower x-axis.

2. Supplementary Tables

Table S1

Name	Units (SI)	Comments
**z**	m	Vertical position. z = 0 corresponds to the bottom of the yeast pillar.
**C_0**	moles.m^{-3}	Concentration far away from the colony.
**C***	moles.m^{-3}	Average concentration below the colony at z = 0
**q**	moles.m^{-3}.s^{-1}	Specific glucose absorption by cells.
**µ**	s^{-1}	Growth rate of single cells
**y**	m^3.$moles^{-1}$	Volume of yeast produced per unit glucose
**γ**	m.s^{-1}	Vertical growth rate of the yeast pillar
**D**	m^2.s-1	Diffusion coefficient of glucose
**H_m**	m	Distance of glucose penetration into the yeast pillar.
**Y**	m^3.$moles^{-1}$	Yield at the scale of the colony. Volume of yeast colony produced per moles glucose.
**J_0**	moles.m^{-2}.s^{-1}	Maximum glucose influx at z=0
**J**	moles.m^{-2}.s^{-1}	Average glucose influx at z=0
**I_0**	moles.s^{-1}	Maximum total glucose flux at z = 0
**I**	moles.s^{-1}	Total glucose flux at z = 0
**A**	m	Radius of the cylindrical colony.
**Name**	**Value**	**Comments**
**D_1**	$6.10^{-10}m^2.s^{-1}$	Typical diffusion coefficient of a small solute in liquid is the same than in 2% Agar gel (8)
**D_2**	$1.44.10^{-10}m^2.s^{-1}$	Measured diffusion of glucose through yeast layers (9)
**q**	Table S7	Depends on glucose concentration
**µ**	Table S7	Depends on glucose concentration
**a**	$0.75.10^{-3}$m	Radius of the yeast pillar.
**R_{gel}**	$2.75.10^{-2}$m	Radius of the Petri dish
**h_{gel}**	$6.3.10^{-3}$m	Depth of the gel(considering a gel volume of 15 mL)

Table S1: Name and numerical values of the variables used in the models and in the text. Note that we did not fit any parameters in our numerical simulations and simply chose parameters that lay within the range of values reported in the literature (Figure S5 and Table S7).

Table S2

**From Youk*et al.*(6)**				
C (mM)	$\mu (h^{-1})$	q (g_{glu}/g_{DW}/h)	q ($mol.s^{-1}m^{-3}$)	Y (g_{DW}/g_{glu})
0.333	0.42	0.38	0.3	1.08
1.11	0.50	1.09	0.86	0.46
3.33	0.54	1.70	1.35	0.31
11.1	0.56	2.15	1.71	0.26
33.3	0.57	2.77	2.2	0.21
55.5	0.59	4.70	3.73	0.12
111	0.59	6.92	5.48	0.08
222	0.59	10.00	7.89	0.06
C>222	0.59	Linear increase	Linear increase	Linear decrease
**From Reifenberger et al.**(5)				
Uptake				
V_{max1}	1.575 $mol.m^{-3}.s^{-1}$	V_{max2}	0.98 $mol.m^{-3}.s^{-1}$	
Km_1	0.8 mM	Km_2	21 mM	
Growth				
μ_{max}	0.31 h^{-1}	C_{min}	1 mM	

Table S2: Glucose consumption and growth rate from Youk *et al.*(6) and data from Reifengerger *et al.* (5). See also Figure S5. These data were extracted manually from the figures in these papers and converted to proper units. This required converting the biomass into dry weight using data from the literature (7) for the cell density (1.1 g/mL) and for the cell dry weight (40% of its wet weight). Although there are some quantitative differences, the qualitative variations are comparable between the different sets of data (see figure S5). For Reifenberger data, the specific glucose uptake rate q was assumed to vary as $q(C) = \frac{V_{max1}C}{Km_1+C} + \frac{V_{max2}C}{Km_2+C}$ and μ was set as $\mu(C) = \mu_{max}\left(1 - e^{(C_{min}-C)}\right)$. C_{min} was chosen by comparison of liquid cultures were no growth could be observed under 1 mM of Glucose.

3. Supplementary movie SM1

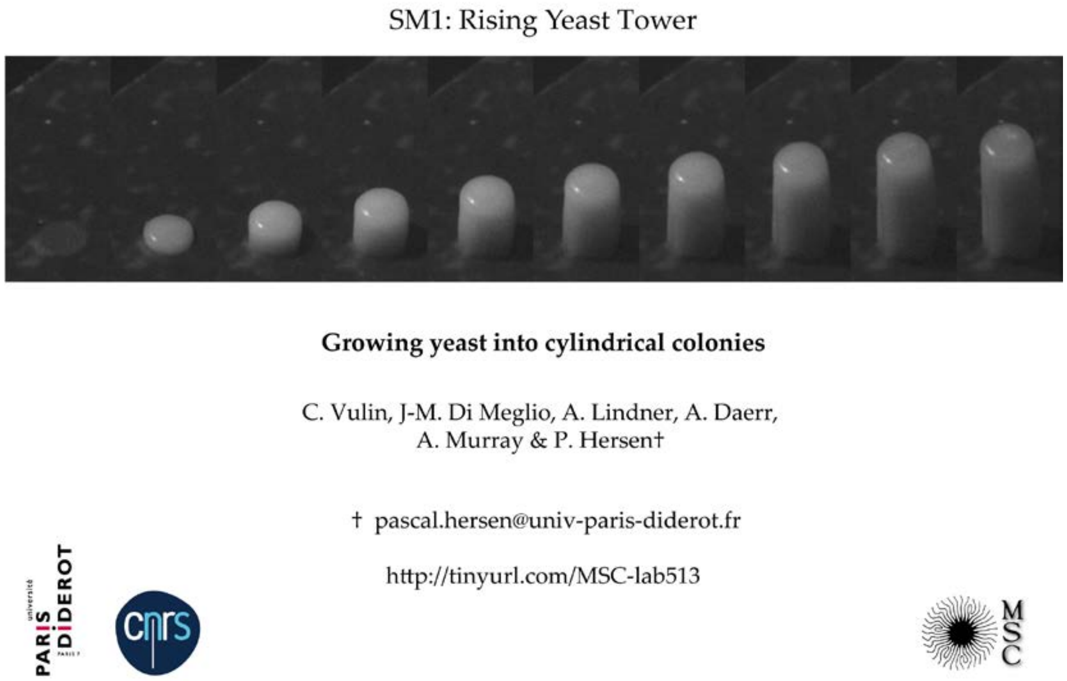

SM1: A *S. cerevisiae* colony is grown on a modified I*sopore*™ membrane patterned with a 1.5 mm diameter porous disk. The colony develops upside down (to avoid buckling) for over two weeks with a constant growth rate. Snapshots of this video are shown in figure 2A.

4. Analytic derivation.

In order to derive a simple analytical model, we start by assuming that the growth of a cylinder can be treated as a one dimensional stationary problem. We thus have to solve:

$$\frac{\partial C}{\partial t} = D\frac{\partial^2 \mathrm{C}}{\partial \mathrm{z}^2} - q(C) \text{ for z} \geq 0$$

We further assume that the system quickly reaches a steady state and that the glucose reservoir is infinite with a concentration C_0 far away from the colony. Crank has proposed a derivation of the total influx, at the level of a perfectly absorbing disk, of a molecule that diffuses with a diffusion coefficient D in a semi-infinite space:

$$I_0 = 4DC_0a = \pi a^2 J_0$$

I_0 is the maximum number of glucose molecules that can be absorbed by our cylindrical colony. In our case, we have to take into account the residual surface concentration C*. We further assume that in such a case the flux (of glucose) absorbed per unit time at the bottom of the colony reads :

$$I = 4DC_0a\,(1 - \frac{C^*}{C_0}) = \pi a^2 J$$

Let's analyze the simple scenario where, the growth rate, μ, the glucose absorptionrate per unit volume and time, q, and the single yeast cell yield, $y = \mu/q$ are independent of the local concentration C.

The diffusion equation can be solved with the following boundary conditions:

$$C(0) = C^*; C(H) = 0; \left.\frac{dC}{dz}\right|_{z=0} = \frac{I}{\pi a^2 D}; \left.\frac{dC}{dz}\right|_{z=H} = 0$$

And since we have a relation between the glucose influx and the surface concentration, it is possible to solve this set of equations as a function of the glucose concentration C_0, q and μ.

$$C^* = C_0 + \frac{\tilde{c}}{2}\left(1 - \sqrt{1 + 4\frac{C_0}{\tilde{c}}}\right)$$

$$I = I_0\frac{\tilde{c}}{2C_0}\left(\sqrt{1 + 4\frac{C_0}{\tilde{c}}} - 1\right)$$

Which gives H:

$$H = \frac{I}{\pi a^2 q}$$

$$\tilde{c} = \frac{\pi^2}{8}\frac{a^2 q}{D}$$

Interestingly, the calculus introduces a typical concentration of the problem, $\tilde{c}$ which depends on q, a and D. The ratio between this typical concentration and C_0 compares the absorption of glucose per unit time in the cylindrical colony over a distance a $(a^3 q)$to the maximum glucose flux that the gel can deliver $(4aDC_0)$. One can also define a typical glucose absorption as:

$$\tilde{q} = \frac{8DC_0}{\pi^2 a^2}$$

This typical glucose absorption rate defines a regime of high glucose absorption $(q \gg \tilde{q})$ where the maximum incoming flux ($4DaC_0$) is small compared to the absorption of glucose within the pilar. For $q \ll \tilde{q}$, the absorption inside the pillar is small compared to what the gel can deliver by diffusion, and thus the surface concentration increases.

The yeast cylinder growth rate is simply given by

$$\frac{dh}{dt} = \gamma = \mu H = \frac{\mu}{q}\frac{I}{\pi a^2}$$

On this expression, one can see that in the ideal case of a perfectly absorbing disk, the glucose influx is limited by the diffusion of glucose in the gel and thus does not depend on the metabolic properties of the yeast cells (I = 4DaC0). As a result, the vertical growth rate is set by the ratio μ/q that is the yield of cells inside the colony. If one compares two strains with similar yield, increasing the glucose absorption, decrease of the thickness growing cells but also speeds up their division (same yield), and thus the two opposing effects cancel each other out. In other words, the growth rate of the colony is set by the metabolic yield and not the single cell division rate. This is why we argue in the main text that this case is fundamentally different than the case of liquid culture, where μ is the only relevant parameter.

Coming back to the solution of our problem, there will be two limiting cases, depending on the value of $\tilde{c}/C_0$. The first limiting case is the one corresponding to a solution close to a perfect absorption, where $\frac{\tilde{c}}{C_0} \gg 1$. Taking the case of C_0 = 111 mM, we have $\tilde{q} \sim 0.1\, mol.m^{-3}.s^{-1}$, which means that for $q \sim 1\, mol.m^{-3}.s^{-1}$, the cylinder can absorb more than what the gel can deliver and the surface concentration should be significantly lower than C_0.

We then have:

$$C^* = \frac{{C_0}^2}{\tilde{c}} = \frac{8{C_0}^2 D}{\pi^2 a^2 q}$$

$$I = I_0\left(1 - \frac{C_0}{\tilde{c}}\right) = 4DaC_0\left(1 - \frac{8C_0 D}{\pi^2 a^2 q}\right)$$

$$H = \frac{4DC_0}{\pi a q}\left(1 - \frac{8C_0 D}{\pi^2 a^2 q}\right)$$

So that to the first order in the glucose concentration, the growth is indeed set by the yield

$$\gamma = \frac{\mu}{q}\frac{4DC_0}{\pi a}$$

The other limiting case occurs for very high C_0 or very low glucose absorption. In such situation, we have $\frac{\tilde{c}}{C_0} \ll 1$ and:

$$C^* = C_0\left(1 - \sqrt{\frac{C_0}{\tilde{c}}}\right) = C_0\left(1 - \sqrt{\frac{8DC_0}{\pi^2 a^2 q}}\right)$$

$$I = 2I_0\sqrt{\frac{\tilde{c}}{C_0}} = 4\pi a^2\sqrt{qDC_0/2}$$

$$H = 4\sqrt{\frac{DC_0}{2q}}$$

$$\gamma = \frac{4\mu}{q}\sqrt{\frac{qDC_0}{2}}$$

This limiting case leads to a different scaling behavior of the cylindrical growth. The surface concentration C* is now close to C_0, the distance of penetration of glucose is set by diffusion with an infinite reservoir, and in that case, the vertical growth rate is not set by the yield. Note that however it still depends on both μ and q. Although this model gives an idea of how the main properties of the yeast cylinder, it has to be taken with caution since it relies on the assumption that μ and q do not vary within the colony. To include such variations, we relied on a model of μ and q as a function of the local concentration and to use numerical simulations to solve the system of equation.

5. Numerical modeling

We used COMSOL Multiphysics 3.5 to solve the time-dependent, non-linear diffusion equation for glucose (equation 1). Glucose was assumed to be the limiting nutrient and to be able to freely diffuse, with two diffusion constants, D_1 in the gel (7) and D_2 in the cylindrical colony (8). Glucose absorption took place into the cylindrical colony only. To do so, we used specific glucose absorption data from Youk *et al*. (5) or Reifenberger *et al.* (4)and interpolate them using piecewise linearization (see Table S1 and S2). To convert their data into a glucose uptake per unit volume we used our experimental measurement (see Figure S4).

We then solve the diffusion equation for glucose in the glucose reservoir and inside the yeast pillar. For simplicity, we started from an already formed cylindrical colony that was high enough (3 mm) so that the glucose concentration will be zero in the upper part of the cylinder. The growth of the cylinder was deduced afterwards integrating single cell growth rate based on computed local concentration.

The set of equations that we solved is:

$$\frac{\partial C}{\partial t} = D_i \Delta C - q(C)\ ;\ (\mathrm{D}_i = \mathrm{D}_1, z < 0, \mathrm{D}_i = \mathrm{D}_2\ \mathrm{z} > 0)(1)$$

$$q(C), \mathrm{z} \geq 0\ ;\ q(C) = 0\ , \mathrm{z} < 0\ \ (2)$$

$$\frac{dh}{dt} = \frac{1}{\pi a^2} \iint \mu\big(\mathrm{c}(\mathrm{r},\mathrm{z},\mathrm{t})\big) 2\pi r dr dz\ (3)$$

We solved this coupled set of equations taking advantage of the cylindrical symmetry. The parameters of the model are the diffusion of glucose (D_1, D_2), the relationship between glucose uptake, *q*, and the glucose concentration, *C*, and the local growth rate, µ,which depends on the local glucose concentration. The radius of the cylinder, *a*, the initial concentration of glucose, C_0, and the dimensions of the Petri dish were similar to that of our experimental system. Numerical simulations were run to reach an equivalent time of 50 hours, with small enough time step (3 sec) and space resolution (a automatically generated triangular grid was set to be at most 1 µm at gel/colony interface) so as to capture correctly the dynamics of the diffusion equation. The tables S1 and S2 summarize the different parameters of our problem.

6. Supporting References

1. Rieger M, Käppeli O, Fiechter A. 1983. The role of Limited Respiration in the incomplete oxidation of glucose by Saccharomyces cerevisiae. *J Gen micobiology* 129:653–661.

2. Postma E, Scheffers WA, van Dijken JP. 1989. Kinetics of growth and glucose transport in glucose-limited chemostat cultures of Saccharomyces cerevisiae CBS 8066. *Yeast* 5:159–165.

3. Diderich, J. a, M. Schepper, P. van Hoek, M. a Luttik, J.P. van Dijken, et al. 1999. Glucose uptake kinetics and transcription of HXT genes in chemostat cultures of Saccharomyces cerevisiae. J. Biol. Chem. 274: 15350–15359.

4. Reifenberger E, Boles E, Ciriacy M. 1997. Kinetic characterization of individual hexose transporters of Saccharomyces cerevisiae and their relation to the triggering mechanism of glucose repression. *Eur J Biochem* 245:324–333.

5. Youk H, van Oudenaarden A. 2009. Growth landscape formed by perception and import of glucose in yeast. *Nature* 462:875–9.

6. Bionumbers http://bionumbers.hms.harvard.edu.

7. Weng L, Liang S, Zhang L, Zhang X, Xu J. 2005. Transport of Glucose and Poly(ethylene glycol)s in Agarose Gels Studied by the Refractive Index Method. *Macromolecules* 38:5236–5242.

8. Converti A, Casagrande M, De Giovanni M, Robatti M, Del Borghi M. 1996. Evaluation of glucose diffusion coefficient through cell layers for the kinetic study of an immobilized cell reactor. *Chem Eng Sci* 51:1032–1026.
